# Supplementary material for: Associations of Plastic Bottle Exposure with Infant Growth, Fecal Microbiota, and Short-Chain Fatty Acids
Source: Microorganisms. 2023 Dec 5;11(12):2924. doi: 10.3390/microorganisms11122924 (PMC10745781; doi:10.3390/microorganisms11122924)
Supplement: Supplementary file 1 [file microorganisms-11-02924-s001.zip › microorganisms-2723833-supplementary.pdf]

		Month 3	Month 12	Trajectory month 3-12
Growth Measures N=442	Subscapular Skinfold	-	N = 360	N = 442
	Abdominal Skinfold	-	N = 358	N = 442
	Triceps Skinfold	-	N = 359	N = 442
	Subscapular + Triceps Skinfold	-	N = 359	N = 442
	Length-for-age	-	N = 359	N = 442
	BMI-for-age	-	N = 359	N = 442
	Weight-for-length	-	N = 359	N = 442
Fecal Measures N=70	Microbiota	N = 62	N = 45	N = 63
	SCFAs	N = 65	N = 46	N = 66

Supplementary Figure S1 [Title]: Flow diagram for the Nurture birth cohort analysis of plastic bottle use frequency at 3 months with anthropometric growth variables, fecal microbiota, and fecal SCFAs.

Supplementary Figure S1 [Legend]: *Of the N=666 infants in the Nurture birth cohort, after exclusion for missing covariate data, there were N=442 with any anthropometric growth measure data at 3, 6, 9, or 12 months of age, and N=70 with any fecal sample measurement at 3 or 12 months of age. Analyses were performed at the 3-month timepoint, at the 12-month timepoint, or by using available data from 3 to 12 months, using the sample sizes above as indicated.*

Abbreviations: SCFA = short chain fatty acids

Supplementary Table S1: Unadjusted and multivariable-adjusted mean differences (95% CIs) in growth parameters assessed at 12 months of age according to plastic bottle use at 3 months of age, among infants from the Nurture birth cohort.

Outcome at 12 Months	Model	Plastic Bottle Use at 3 Months			
		Plastic Bottle Provided Every Feeding	Plastic Bottle Provided 4+/day	Plastic Bottle Provided 1-3/day	Plastic Bottle Provided <1/day
Subscapular Skinfold		N = 231	N = 36	N = 54	N = 39
	M1	-	-0.27 (-0.75, 0.20)	-0.11 (-0.52, 0.31)	-0.38 (-0.87, 0.12)
	M2	-	-0.19 (-0.69, 0.30)	-0.07 (-0.50, 0.36)	-0.31 (-0.81, 0.19)
	M3	-	-0.15 (-0.84, 0.54)	-0.03 (-0.58, 0.51)	-0.28 (-0.83, 0.27)
Abdominal Skinfold		N = 230	N = 36	N = 53	N = 39
	M1	-	0.40 (-0.28, 1.08)	0.08 (-0.52, 0.68)	-0.07 (-0.78, 0.63)
	M2	-	0.42 (-0.28, 1.13)	0.07 (-0.55, 0.69)	0.01 (-0.70, 0.72)
	M3	-	0.66 (-0.32, 1.63)	0.23 (-0.55, 1.01)	0.09 (-0.69, 0.88)
Triceps Skinfold		N = 230	N = 36	N = 54	N = 39
	M1	-	0.23 (-0.45, 0.90)	-0.04 (-0.63, 0.55)	-0.13 (-0.83, 0.57)
	M2	-	0.11 (-0.59, 0.82)	-0.13 (-0.75, 0.48)	-0.16 (-0.87, 0.55)
	M3	-	0.69 (-0.28, 1.67)	0.30 (-0.47, 1.08)	0.15 (-0.63, 0.93)
Subscapular + Triceps Skinfold		N = 230	N = 36	N = 54	N = 39
	M1	-	-0.04 (-1.06, 0.99)	-0.13 (-1.03, 0.76)	-0.50 (-1.55, 0.56)
	M2	-	-0.06 (-1.13, 1.00)	-0.20 (-1.12, 0.73)	-0.46 (-1.54, 0.61)
	M3	-	0.55 (-0.92, 2.02)	0.27 (-0.90, 1.44)	-0.13 (-1.31, 1.05)
BMI-for-age z-score		N = 231	N = 36	N = 53	N = 39
	M1	-	-0.14 (-0.49, 0.21)	0.03 (-0.27, 0.34)	-0.33 (-0.69, 0.03)
	M2	-	-0.14 (-0.49, 0.21)	-0.05 (-0.35, 0.26)	-0.31 (-0.66, 0.05)
	M3	-	-0.02 (-0.50, 0.47)	0.04 (-0.35, 0.43)	-0.26 (-0.65, 0.13)
Length-for-age z-score		N = 231	N = 36	N = 53	N = 39
	M1	-	-0.20 (-0.55, 0.16)	**-0.38 (-0.70, -0.07)**	0.06 (-0.31, 0.43)
	M2	-	-0.25 (-0.61, 0.12)	**-0.45 (-0.76, -0.13)**	0.08 (-0.29, 0.44)
	M3	-	0.20 (-0.30, 0.69)	-0.17 (-0.56, 0.23)	0.22 (-0.18, 0.62)
Weight-for-length z-score		N = 231	N = 36	N = 53	N = 39
	M1	-	-0.17 (-0.51, 0.18)	-0.02 (-0.33, 0.28)	-0.31 (-0.67, 0.05)

	M2	-	-0.17 (-0.52, 0.17)	-0.11 (-0.41, 0.20)	-0.29 (-0.64, 0.06)
	M3	-	0.02 (-0.46, 0.50)	0.02 (-0.36, 0.40)	-0.22 (-0.61, 0.16)

Table S1 [Footnote]: *Estimates (95% CI) were based on multivariable linear regression models with infant growth parameters as the dependent variables; models were analyzed separately by growth parameters, and complete case analysis was performed for each growth parameter to maximize sample sizes for analyses (sample sizes provided in table).* ***Bold text*** *indicates statistical significance at p<0.05. Models were analyzed according to the following schema:*

M1: unadjusted

M2: M1 + birth weight (kg), gestational age (weeks), maternal age (years), household income (<$20 000 per year vs. ≥ $20 000 per year)

M3: M2 + duration of exclusive breast milk exposure (months), current feeding status (exclusive breast milk, exclusive formula, or mixed feeding)

Abbreviations: CI = confidence interval

Supplementary Table S2: Multivariable-adjusted mean difference (95% CI) in growth parameters assessed at 12 months of age according to plastic bottle use at 3 months of age among subgroups of infants from the Nurture birth cohort.

Outcome at 12 Months	Model	Plastic Bottle Use at 3 Months			
		Plastic Bottle Provided Every Feeding	Plastic Bottle Provided 4+/day	Plastic Bottle Provided 1-3/day	Plastic Bottle Provided <1/day
Subscapular Skinfold (mm)	S1	-	-0.17 (-0.92, 0.59)	-0.03 (-0.64, 0.57)	-0.08 (-0.68, 0.52)
	S2	-	-0.42 (-1.20, 0.37)	-0.10 (-0.71, 0.51)	-0.33 (-0.94, 0.29)
	S3	-	-0.12 (-0.99, 0.75)	-0.06 (-0.76, 0.65)	-0.08 (-0.81, 0.64)
	S4	-	-0.15 (-0.83, 0.52)	-0.02 (-0.57, 0.54)	-0.06 (-0.66, 0.55)
Abdominal Skinfold (mm)	S1	-	0.34 (-0.74, 1.41)	-0.01 (-0.88, 0.85)	0.22 (-0.64, 1.09)
	S2	-	0.52 (-0.66, 1.69)	0.17 (-0.74, 1.08)	-0.20 (-1.12, 0.72)
	S3	-	0.44 (-0.80, 1.69)	0.02 (-1.01, 1.04)	0.24 (-0.80, 1.27)
	S4	-	0.63 (-0.31, 1.56)	0.18 (-0.60, 0.96)	0.43 (-0.41, 1.26)
Triceps Skinfold (mm)	S1	-	0.51 (-0.60, 1.62)	0.09 (-0.81, 0.99)	0.43 (-0.46, 1.32)
	S2	-	1.01 (-0.21, 2.23)	0.46 (-0.48, 1.41)	0.16 (-0.80, 1.11)
	S3	-	0.87 (-0.40, 2.14)	0.36 (-0.67, 1.39)	0.18 (-0.88, 1.24)
	S4	-	0.81 (-0.14, 1.77)	0.47 (-0.33, 1.26)	0.61 (-0.25, 1.47)
Subscapular + Triceps Skinfold (mm)	S1	-	0.34 (-1.31, 2.00)	0.05 (-1.28, 1.39)	0.35 (-0.97, 1.68)
	S2	-	0.59 (-1.19, 2.37)	0.37 (-1.02, 1.75)	-0.17 (-1.57, 1.22)
	S3	-	0.74 (-1.14, 2.63)	0.31 (-1.22, 1.84)	0.10 (-1.48, 1.67)
	S4	-	0.66 (-0.77, 2.09)	0.45 (-0.73, 1.63)	0.55 (-0.73, 1.83)
BMI-for-age z-score	S1	-	-0.15 (-0.69, 0.38)	-0.01 (-0.44, 0.42)	-0.17 (-0.60, 0.26)
	S2	-	0.15 (-0.42, 0.72)	0.13 (-0.32, 0.57)	-0.04 (-0.49, 0.41)
	S3	-	-0.11 (-0.71, 0.50)	-0.15 (-0.64, 0.35)	-0.22 (-0.72, 0.28)
	S4	-	-0.09 (-0.58, 0.39)	0.01 (-0.39, 0.42)	-0.18 (-0.61, 0.25)
Length-for-age z-score	S1	-	0.23 (-0.34, 0.80)	-0.13 (-0.59, 0.33)	0.12 (-0.34, 0.58)
	S2	-	0.28 (-0.33, 0.89)	0.04 (-0.44, 0.51)	-0.03 (-0.51, 0.45)
	S3	-	-0.12 (-0.72, 0.48)	-0.32 (-0.81, 0.17)	-0.26 (-0.75, 0.24)
	S4	-	0.18 (-0.32, 0.68)	-0.22 (-0.63, 0.20)	0.24 (-0.21, 0.69)
Weight-for-length z-score	S1	-	-0.11 (-0.64, 0.42)	-0.03 (-0.45, 0.40)	-0.15 (-0.57, 0.28)
	S2	-	0.20 (-0.37, 0.76)	0.14 (-0.30, 0.57)	-0.04 (-0.49, 0.40)
	S3	-	-0.12 (-0.71, 0.47)	-0.18 (-0.66, 0.30)	-0.25 (-0.74, 0.24)
	S4	-	-0.06 (-0.54, 0.42)	-0.02 (-0.42, 0.39)	-0.14 (-0.57, 0.29)

Table S2 [Footnote]: *All models adjusted for birth weight (kg), gestational age (weeks), maternal age (years), household income (<$20 000 per year vs. ≥ $20 000 per year), duration of exclusive breastfeeding (months), and feeding status at 3 months (exclusive breastmilk, exclusive formula, or mixed feeding). Models were analyzed in the following subgroups:*

S1: among infants whose mothers are not smokers at 3 months.

S2: among infants whose mothers did not take antibiotics during pregnancy.
S3: among infants whose mothers have a post-secondary educational attainment.
S4: among infants who had any breast milk exposure at 3 months.
Sample sizes for each subgroup analysis and outcome are provided in Supplementary Table S3.
Abbreviations: CI = confidence interval

Supplementary Table S3: Sample sizes for subgroup analyses (presented in Supplementary Table S2) assessing the mean difference in 12-month growth parameters according to plastic bottle use at 3 months of age, among subgroups of infants from the Nurture birth cohort.

Outcome at 12 Months	Model	Plastic Bottle Use at 3 Months			
		Plastic Bottle Provided Every Feeding	Plastic Bottle Provided 4+/day	Plastic Bottle Provided 1-3/day	Plastic Bottle Provided <1/day
Subscapular Skinfold	S1	N = 138	N = 30	N = 46	N = 34
	S2	N = 155	N = 25	N = 44	N = 21
	S3	N = 106	N = 24	N = 44	N = 31
	S4	N = 67	N = 31	N = 52	N = 39
Abdominal Skinfold	S1	N = 138	N = 30	N = 46	N = 34
	S2	N = 155	N = 25	N = 44	N = 21
	S3	N = 105	N = 24	N = 43	N = 31
	S4	N = 67	N = 31	N = 51	N = 39
Triceps Skinfold	S1	N = 138	N = 30	N = 46	N = 34
	S2	N = 155	N = 25	N = 44	N = 21
	S3	N = 106	N = 24	N = 44	N = 31
	S4	N = 67	N = 31	N = 52	N = 39
Subscapular + Triceps Skinfold	S1	N = 138	N = 30	N = 46	N = 34
	S2	N = 155	N = 25	N = 44	N = 21
	S3	N = 106	N = 24	N = 44	N = 31
	S4	N = 67	N = 31	N = 52	N = 39
BMI-for-age z-score	S1	N = 138	N = 30	N = 46	N = 34
	S2	N = 155	N = 25	N = 44	N = 21
	S3	N = 106	N = 24	N = 43	N = 31
	S4	N = 67	N = 31	N = 51	N = 39
Length-for-age z-score	S1	N = 138	N = 30	N = 46	N = 34
	S2	N = 155	N = 25	N = 44	N = 21
	S3	N = 106	N = 24	N = 43	N = 31
	S4	N = 67	N = 31	N = 51	N = 39
Weight-for-length z-score	S1	N = 138	N = 30	N = 46	N = 34
	S2	N = 155	N = 25	N = 44	N = 21
	S3	N = 106	N = 24	N = 43	N = 31
	S4	N = 67	N = 31	N = 51	N = 39

Table S3 [Footnote]: *All models adjusted for birth weight (kg), gestational age (weeks), maternal age (years), household income (<$20 000 per year vs. ≥ $20 000 per year), duration of exclusive breastfeeding (months), and feeding status at 3 months (exclusive breastmilk, exclusive formula, or mixed feeding). Models were analyzed in the following subgroups:*

S1: among infants whose mothers are not smokers at 3 months.
S2: among infants whose mothers did not take antibiotics during pregnancy.
S3: among infants whose mothers have a post-secondary educational attainment.
S4: among infants who had any breast milk exposure at 3 months.

Effect estimates for each subgroup analysis and outcome are provided in Supplementary Table S2.

Supplementary Table S4: Multivariable-adjusted mean difference (95% CI) in growth parameters assessed at 12 months of age according to exclusive formula feeding vs. mixed formula and breastmilk feeding at 3 months of age, among the subgroup of infants who are plastic bottle fed at every feeding in the Nurture birth cohort.

Outcome at 12 Months	Exclusive formula feeding at 3 Months	Mixed formula/breastmilk feeding at 3 Months
Subscapular Skinfold	N = 164	N = 66
	-	-0.15 (-0.58, 0.29)
Abdominal Skinfold	N = 163	N = 66
	-	-0.25 (-0.87, 0.37)
Triceps Skinfold	N = 163	N = 66
	-	-0.61 (-1.23, 0.00)
Subscapular + Triceps Skinfold	N = 163	N = 66
	-	-0.75 (-1.69, 0.20)
BMI-for-age z-score	N = 164	N = 66
	-	-0.10 (-0.41, 0.21)
Length-for-age z-score	N = 164	N = 66
	-	-0.11 (-0.41, 0.19)
Weight-for-length z-score	N = 164	N = 66
	-	-0.12 (-0.43, 0.19)

Table S4 [Footnote]: *All models adjusted for birth weight (kg), gestational age (weeks), maternal age (years), household income (<$20 000 per year vs. ≥ $20 000 per year), and duration of exclusive breastfeeding (months).*
Abbreviations: CI = confidence interval

Supplementary Table S5: Distribution of anthropometric growth measures in infants from the Nurture birth cohort at 3, 6, 9, and 12 months after birth, stratified by plastic bottle feeding frequency at 3 months of age.

	Every feeding (N=299)				Less than every feeding (N=143)			
	Month 3	Month 6	Month 9	Month 12	Month 3	Month 6	Month 9	Month 12
**Subscapular Skinfolds (mm)**								
Mean (SD)	7.83 (1.66)	7.76 (1.79)	7.51 (1.69)	7.22 (1.48)	7.74 (1.73)	7.54 (1.54)	7.35 (1.23)	6.99 (1.24)
Missing	1 (0.3%)	50 (16.7%)	68 (22.7%)	68 (22.7%)	2 (1.4%)	15 (10.5%)	18 (12.6%)	14 (9.8%)
**Abdominal Skinfolds (mm)**								
Mean (SD)	8.73 (2.30)	8.34 (2.42)	7.57 (2.10)	7.26 (2.12)	8.79 (2.26)	8.56 (2.13)	7.91 (1.92)	7.39 (1.77)
Missing	3 (1.0%)	50 (16.7%)	68 (22.7%)	69 (23.1%)	2 (1.4%)	18 (12.6%)	19 (13.3%)	15 (10.5%)
**Triceps Skinfolds (mm)**								
Mean (SD)	8.95 (1.85)	9.51 (2.27)	9.09 (2.22)	8.82 (2.10)	8.83 (1.82)	9.46 (2.03)	9.08 (1.85)	8.84 (1.74)
Missing	1 (0.3%)	50 (16.7%)	69 (23.1%)	69 (23.1%)	2 (1.4%)	15 (10.5%)	18 (12.6%)	14 (9.8%)
**Subscapular+Triceps Skinfolds (mm)**								
Mean (SD)	16.8 (3.15)	17.3 (3.61)	16.6 (3.50)	16.0 (3.20)	16.6 (3.09)	17.0 (3.04)	16.4 (2.64)	15.8 (2.58)
Missing	1 (0.3%)	50 (16.7%)	69 (23.1%)	69 (23.1%)	3 (2.1%)	15 (10.5%)	18 (12.6%)	14 (9.8%)
**Length-for-age z-score**								
Mean (SD)	-0.417 (1.09)	-0.0965 (1.04)	-0.210 (1.20)	-0.173 (1.04)	-0.190 (1.02)	-0.204 (1.05)	-0.280 (1.11)	-0.376 (1.07)
Missing	1 (0.3%)	51 (17.1%)	71 (23.7%)	68 (22.7%)	0 (0%)	15 (10.5%)	19 (13.3%)	15 (10.5%)
**BMI-for-age z-score**								
Mean (SD)	-0.00262 (1.07)	0.302 (1.16)	0.575 (1.14)	0.739 (1.08)	-0.0550 (1.02)	0.277 (0.935)	0.489 (0.813)	0.617 (0.911)
Missing	1 (0.3%)	51 (17.1%)	72 (24.1%)	68 (22.7%)	0 (0%)	15 (10.5%)	19 (13.3%)	15 (10.5%)
**Weight-for-length z-score**								
Mean (SD)	0.159 (1.07)	0.399 (1.15)	0.605 (1.11)	0.699 (1.08)	0.0489 (1.02)	0.381 (0.917)	0.518 (0.807)	0.550 (0.889)
Missing	1 (0.3%)	51 (17.1%)	72 (24.1%)	68 (22.7%)	0 (0%)	15 (10.5%)	19 (13.3%)	15 (10.5%)

Table S5 [Footnote]: *Distribution of anthropometric growth measures at 3, 6, 9, and 12 months of age are provided for N=442 infants from the Nurture birth cohort, stratified by plastic bottle use frequency at 3 months (dichotomized as plastic bottle fed at every feeding vs. plastic bottle*

fed less than every feeding). Growth measures are presented as mean (SD) and missingness for each timepoint is presented as N (%).
Abbreviations: SD = standard deviation

Supplementary Table S6: Unadjusted and multivariable-adjusted mean differences (95% CIs) in fecal microbiota alpha diversity measures (assessed at 3 months and 12 months of age) according to plastic bottle use at 3 months of age, among infants from the Nurture birth cohort.

Outcome		3 months		12 months	
		Plastic Bottle Provided Every Feeding	Plastic Bottle Provided Less Than Every Feeding	Plastic Bottle Provided Every Feeding	Plastic Bottle Provided Less Than Every Feeding
		N= 48	N= 14	N= 34	N= 11
Observed ASVs	M1	-	**-16.60 (-27.08, -6.12)**	-	-16.65 (-35.65, 2.36)
	M2	-	**-23.65 (-39.33, -7.96)**	-	-17.26 (-41.47, 6.96)
	M3	-	-15.12 (-35.15, 4.92)	-	-20.45 (-56.37, 15.46)
Pielou Evenness	M1	-	**-0.07 (-0.12, -0.01)**	-	0.00 (-0.06, 0.07)
	M2	-	**-0.09 (-0.17, -0.01)**	-	-0.01 (-0.10, 0.08)
	M3	-	-0.06 (-0.16, 0.03)	-	-0.03 (-0.16, 0.11)
Shannon Diversity	M1	-	**-0.43 (-0.70, -0.17)**	-	-0.08 (-0.47, 0.30)
	M2	-	**-0.59 (-0.99, -0.20)**	-	-0.15 (-0.65, 0.35)
	M3	-	-0.45 (-0.92, 0.03)	-	-0.22 (-0.97, 0.52)

Table S6 [Footnote]: *Estimates (95% CI) were based on multivariable linear regression models with fecal microbiota alpha diversity measures as the dependent variables; models were analyzed separately by alpha diversity measure and by timepoint.* ***Bold text*** *indicates statistical significance at p<0.05. Models were analyzed according to the following schema:*

M1: unadjusted

M2: M1 + birth weight (kg), gestational age (weeks), maternal age (years), household income (<$20 000 per year vs. ≥ $20 000 per year)

M3: M2 + duration of exclusive breast milk exposure (months), current feeding status (exclusive breast milk, exclusive formula, or mixed feeding)

Observed ASV models additionally adjust for unrarefied sequencing depth in all models M1-M3.

Abbreviations: ASV = amplicon sequencing variant, CI = confidence interval

Supplementary Table S7: Multivariable-adjusted mean differences (95% CIs) in fecal microbiota alpha diversity measures (assessed at 3 months and 12 months of age) according to plastic bottle use at 3 months of age, among subgroups of infants from the Nurture birth cohort.

Outcome		3 months		12 months	
		Plastic Bottle Provided Every Feeding	Plastic Bottle Provided Less Than Every Feeding	Plastic Bottle Provided Every Feeding	Plastic Bottle Provided Less Than Every Feeding
Observed ASVs	S1	-	-7.41 (-33.98, 19.17)	-	-15.50 (-58.70, 27.70)
	S2	-	**-24.82 (-48.74, -0.89)**	-	-20.35 (-63.59, 22.90)
	S3	-	-24.47 (-57.93, 8.99)	-	-14.47 (-56.77, 27.83)
	S4	-	**-27.28 (-52.46, -2.11)**	-	2.40 (-64.24, 69.04)
Pielou Evenness	S1	-	-0.01 (-0.12, 0.09)	-	0.00 (-0.16, 0.16)
	S2	-	-0.06 (-0.16, 0.04)	-	-0.01 (-0.16, 0.15)
	S3	-	-0.07 (-0.23, 0.08)	-	-0.01 (-0.17, 0.16)
	S4	-	-0.06 (-0.20, 0.09)	-	0.06 (-0.15, 0.27)
Shannon Diversity	S1	-	-0.17 (-0.72, 0.38)	-	-0.05 (-0.94, 0.83)
	S2	-	-0.50 (-1.00, 0.01)	-	-0.14 (-1.03, 0.76)
	S3	-	-0.59 (-1.35, 0.16)	-	-0.10 (-1.03, 0.83)
	S4	-	-0.53 (-1.17, 0.12)	-	0.27 (-0.89, 1.42)

Table S7 [Footnote]: *All models adjusted for birth weight (kg), gestational age (weeks), maternal age (years), household income (<$20 000 per year vs. ≥ $20 000 per year), duration of exclusive breastfeeding (months), and feeding status at 3 months (exclusive breastmilk, exclusive formula, or mixed feeding). Observed ASV models were additionally adjusted for unrarefied sequencing depth. Models were analyzed in the following subgroups:*

S1: among infants whose mothers are not smokers at 3 months.

S2: among infants whose mothers did not take antibiotics during pregnancy.

S3: among infants whose mothers have a post-secondary educational attainment.

S4: among infants who had any breast milk exposure at 3 months.

Sample sizes for each subgroup analysis and outcome are provided in Supplementary Table S8.

Abbreviations: ASV = amplicon sequencing variant, CI = confidence interval

Supplementary Table S8: Sample sizes for subgroup analyses (presented in Supplementary Table S7) assessing the mean difference in fecal microbiota alpha diversity metrics (measured at 3 months and 12 months of age) according to plastic bottle use at 3 months of age, among subgroups of infants from the Nurture birth cohort.

Model	3 months		12 months	
	Plastic Bottle Provided Every Feeding	Plastic Bottle Provided Less Than Every Feeding	Plastic Bottle Provided Every Feeding	Plastic Bottle Provided Less Than Every Feeding
S1	N = 21	N = 12	N = 17	N = 10
S2	N = 34	N = 12	N = 23	N = 10
S3	N = 19	N = 13	N = 13	N = 10
S4	N = 13	N = 11	N = 6	N = 10

Table S8 [Footnote]: *All models adjusted for birth weight (kg), gestational age (weeks), maternal age (years), household income (<$20 000 per year vs. ≥ $20 000 per year), duration of exclusive breastfeeding (months), and feeding status at 3 months (exclusive breastmilk, exclusive formula, or mixed feeding). Observed ASV models were additionally adjusted for unrarefied sequencing depth. Models were analyzed in the following subgroups:*

S1: among infants whose mothers are not smokers at 3 months.

S2: among infants whose mothers did not take antibiotics during pregnancy.

S3: among infants whose mothers have a post-secondary educational attainment.

S4: among infants who had any breast milk exposure at 3 months.

Effect estimates for each subgroup analysis and outcome are provided in Supplementary Table S7.

Supplementary Table S9: Multivariable-adjusted mean difference (95% CI) in fecal microbiota alpha diversity metrics (assessed at 3 months and 12 months of age) according to exclusive formula feeding vs. mixed formula and breastmilk feeding at 3 months of age, among the subgroup of infants who are plastic bottle fed at every feeding in the Nurture birth cohort.

Outcome	Fecal microbiota at 3 months		Fecal microbiota at 12 months	
	Exclusive formula feeding at 3 months	Mixed formula/breastmilk feeding at 3 months	Exclusive formula feeding at 3 months	Mixed formula/breastmilk feeding at 3 months
	N=35	N=13	N=28	N=6
Observed ASVs	-	6.70 (-5.85, 19.25)	-	26.53 (-1.76, 54.81)
Pielou Evenness	-	0.04 (-0.02, 0.10)	-	0.05 (-0.07, 0.17)
Shannon Diversity	-	0.25 (-0.08, 0.58)	-	0.36 (-0.21, 0.93)

Table S9 [Footnote]: *All models adjusted for birth weight (kg), gestational age (weeks), maternal age (years), household income (<$20 000 per year vs. ≥ $20 000 per year), and duration of exclusive breastfeeding (months). Observed ASVs were additionally adjusted for unrarefied sequencing depth. Abbreviations: ASV = amplicon sequencing variant, CI = confidence interval*

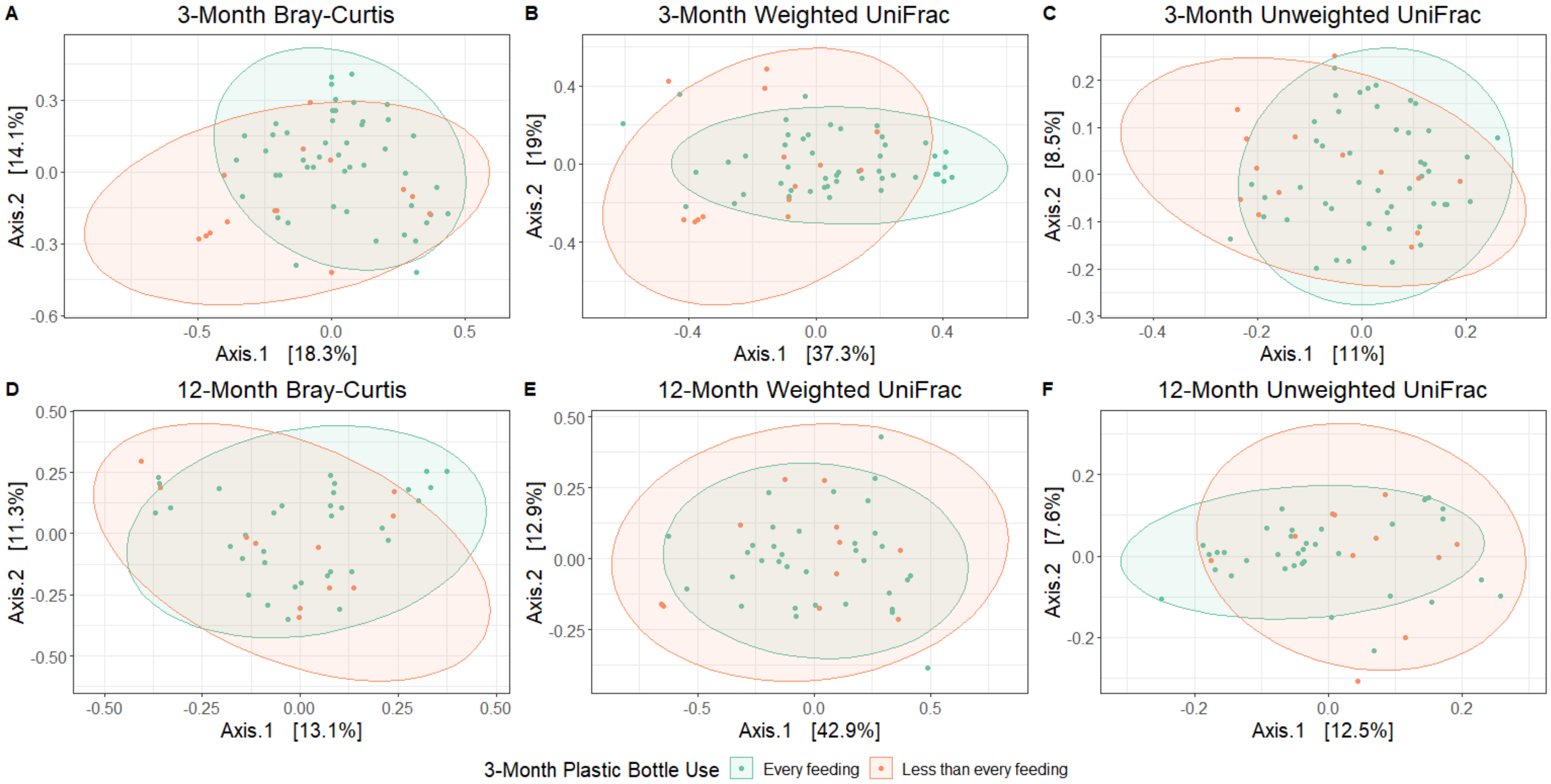

Supplementary Figure S2 [Title]: Principal coordinate analysis plots for fecal microbiota beta diversity metrics (measured at 3 months and at 12 months of age) by 3-month plastic bottle use frequency, among infants from the Nurture birth cohort.

Supplementary Figure S2 [Legend]: *A total of N=62 infants (for 3 months microbiota samples) and N=45 (for 12 months microbiota samples) were included in the plots. Points represent individual infant microbiota samples and are color-coded by plastic bottle use frequency at 3 months of age; ellipses represent the 95% confidence level for a multivariate t-distribution for each plastic bottle frequency grouping. Clustering of samples depicts greater similarity in microbiota compositions based on the beta diversity metric.*

*Panels: (**A**) Bray-Curtis dissimilarity, at 3 months of age. (**B**) Weighted UniFrac distance, at 3 months of age. (**C**) Unweighted UniFrac distance, at 3 months of age. (**D**) Bray-Curtis dissimilarity, at 12 months of age. (**E**) Weighted UniFrac distance, at 12 months of age. (**F**) Unweighted UniFrac distance, at 12 months of age.*

Supplementary Table S10: The amount of variance (adjusted R^2) in fecal microbiota beta diversity metrics (measured at 3 months and 12 months of age) attributed to the frequency of plastic bottle use at 3 months of age, in infants from the Nurture birth cohort.

Month	Sample Size	Model	Bray-Curtis		Unweighted UniFrac		Weighted UniFrac	
			Adjusted R^2	p-value	Adjusted R^2	p-value	Adjusted R^2	p-value
3	N=62	M1	**0.03999**	**0.0032**	**0.03084**	**0.0037**	**0.05636**	**0.0050**
		M2	0.02462	0.0836	**0.02720**	**0.0117**	0.03416	0.0502
		M3	0.01013	0.8714	0.01982	0.1609	0.01461	0.4201
12	N=45	M1	0.01896	0.7093	0.02630	0.2122	0.01113	0.8299
		M2	0.01880	0.7481	0.02596	0.2512	0.01011	0.8849
		M3	0.02024	0.6641	0.02218	0.5309	0.01604	0.6378

Table S10 [Footnote]: *Adjusted R^2 and p-values are based on permutational analysis of variance (PERMANOVA) models with fecal microbiota beta diversity measures as the dependent variables; models were analyzed separately by beta diversity measure and by timepoint, with 9 999 permutations. Measures reflect the contribution of plastic bottle use frequency at 3 months of age (dichotomized as plastic bottle fed at every feeding vs. plastic bottle fed less than every feeding) to the variance in each beta diversity metric.* ***Bold text*** *indicates statistical significance at $p<0.05$. Models were analyzed according to the following schema:*

M1: unadjusted

M2: M1 + birth weight (kg), gestational age (weeks), maternal age (years), household income (<$20 000 per year vs. ≥ $20 000 per year)

M3: M2 + duration of exclusive breast milk exposure (months), current feeding status (exclusive breast milk, exclusive formula, or mixed feeding)

Abbreviations: CI = confidence interval, PERMANOVA = permutational analysis of variance

Supplementary Table S11: Multivariable-adjusted mean differences (95% CIs) in fecal SCFAs (assessed at 3 months and 12 months of age) according to plastic bottle use at 3 months of age, among subgroups of infants from the Nurture birth cohort.

Outcome		3 months		12 months	
		Plastic Bottle Provided Every Feeding	Plastic Bottle Provided Less Than Every Feeding	Plastic Bottle Provided Every Feeding	Plastic Bottle Provided Less Than Every Feeding
Total SCFAs	S1	-	-3.09 (-57.86, 51.69)	-	-16.58 (-94.85, 61.69)
	S2	-	21.04 (-32.35, 74.42)	-	-42.03 (-109.29, 25.22)
	S3	-	-9.79 (-71.14, 51.56)	-	-41.57 (-127.86, 44.71)
	S4	-	35.89 (-52.63, 124.41)	-	-8.12 (-166.24, 150.00)
Acetic acid	S1	-	0.45 (-39.63, 40.53)	-	-19.00 (-80.06, 42.07)
	S2	-	25.82 (-12.98, 64.62)	-	-30.12 (-117.29, 57.05)
	S3	-	2.45 (-43.46, 48.35)	-	-26.04 (-82.56, 30.48)
	S4	-	31.85 (-32.18, 95.87)	-	-14.89 (-120.83, 91.04)
Propionic acid	S1	-	0.16 (-8.59, 8.90)	-	6.75 (-6.66, 20.16)
	S2	-	-2.05 (-13.28, 9.18)	-	-4.08 (-22.75, 14.59)
	S3	-	-4.35 (-16.94, 8.25)	-	-7.06 (-38.44, 24.31)
	S4	-	1.64 (-21.00, 24.27)	-	7.08 (-32.51, 46.66)
Butyric acid	S1	-	-2.26 (-11.89, 7.37)	-	-4.89 (-21.45, 11.68)
	S2	-	-1.28 (-10.95, 8.39)	-	-10.26 (-25.83, 5.31)
	S3	-	-5.45 (-14.9, 4.00)	-	-10.92 (-34.06, 12.22)
	S4	-	0.49 (-11.77, 12.75)	-	-4.53 (-41.46, 32.41)
Isobutyric acid	S1	-	-0.72 (-3.36, 1.92)	-	-0.42 (-2.97, 2.12)
	S2	-	-0.02 (-2.35, 2.30)	-	0.63 (-1.52, 2.77)
	S3	-	-0.15 (-2.57, 2.27)	-	0.41 (-1.79, 2.61)
	S4	-	1.03 (-1.13, 3.19)	-	0.67 (-3.88, 5.22)
Valeric acid	S1	-	-0.08 (-1.96, 1.80)	-	0.98 (-0.66, 2.63)
	S2	-	-0.42 (-2.48, 1.64)	-	1.18 (-1.80, 4.15)
	S3	-	-0.30 (-2.40, 1.79)	-	1.99 (-0.42, 4.39)
	S4	-	0.02 (-0.94, 0.98)	-	0.13 (-3.25, 3.51)
Isovaleric acid	S1	-	-0.70 (-2.83, 1.42)	-	-0.07 (-2.32, 2.18)
	S2	-	-1.15 (-3.24, 0.94)	-	0.57 (-1.66, 2.81)
	S3	-	-1.73 (-4.11, 0.65)	-	0.02 (-1.29, 1.32)
	S4	-	1.04 (-2.23, 4.32)	-	3.32 (-0.57, 7.21)
Hexanoic acid	S1	-	0.07 (-0.73, 0.86)	-	0.06 (-0.52, 0.65)
	S2	-	0.14 (-0.61, 0.88)	-	0.05 (-0.29, 0.39)
	S3	-	-0.26 (-1.23, 0.72)	-	0.04 (-0.52, 0.60)
	S4	-	-0.17 (-1.23, 0.88)	-	0.10 (-0.68, 0.89)

Table S11 [Footnote]: *All models adjusted for birth weight (kg), gestational age (weeks), maternal age (years), household income (<$20 000 per year vs. ≥ $20 000 per year), duration of exclusive breastfeeding (months), and feeding status at 3 months (exclusive breastmilk, exclusive formula, or mixed feeding). Models were analyzed in the following subgroups:*

S1: among infants whose mothers are not smokers at 3 months.

S2: among infants whose mothers did not take antibiotics during pregnancy.
S3: among infants whose mothers have a post-secondary educational attainment.
S4: among infants who had any breast milk exposure at 3 months.
Sample sizes for each subgroup analysis and outcome are provided in Supplementary Table S12.
Abbreviations: CI = confidence interval, SCFA = short chain fatty acid

Supplementary Table S12: Sample sizes for subgroup analyses (presented in Supplementary Table S11) assessing the mean difference in fecal SCFAs (measured at 3 months and 12 months of age) according to plastic bottle use at 3 months of age, among subgroups of infants from the Nurture birth cohort.

Model	3 months		12 months	
	Plastic Bottle Provided Every Feeding	Plastic Bottle Provided Less Than Every Feeding	Plastic Bottle Provided Every Feeding	Plastic Bottle Provided Less Than Every Feeding
S1	N = 22	N = 12	N = 17	N = 10
S2	N = 37	N = 12	N = 24	N = 10
S3	N = 21	N = 13	N = 13	N = 10
S4	N = 14	N = 11	N = 6	N = 10

Table S12 [Footnote]: *All models adjusted for birth weight (kg), gestational age (weeks), maternal age (years), household income (<$20 000 per year vs. ≥ $20 000 per year), duration of exclusive breastfeeding (months), and feeding status at 3 months (exclusive breastmilk, exclusive formula, or mixed feeding). Models were analyzed in the following subgroups:*

S1: among infants whose mothers are not smokers at 3 months.
S2: among infants whose mothers did not take antibiotics during pregnancy.
S3: among infants whose mothers have a post-secondary educational attainment.
S4: among infants who had any breast milk exposure at 3 months.

Effect estimates for each subgroup analysis and outcome are provided in Supplementary Table S11.

Supplementary Table S13: Multivariable-adjusted mean difference (95% CI) in SCFAs (assessed at 3 months and 12 months of age) according to exclusive formula feeding vs. mixed formula and breastmilk feeding at 3 months of age, among the subgroup of infants who are plastic bottle fed at every feeding in the Nurture birth cohort.

Outcome	3 months		12 months	
	Exclusive formula feeding	Mixed formula/breastmilk feeding	Exclusive formula feeding	Mixed formula/breastmilk feeding
	N=37	N=14	N=29	N=6
Total SCFAs	-	5.07 (-28.96, 39.10)	-	21.61 (-59.24, 102.47)
Acetic acid	-	11.29 (-13.27, 35.85)	-	4.93 (-50.19, 60.04)
Propionic acid	-	0.45 (-6.27, 7.18)	-	4.74 (-7.23, 16.72)
Butyric acid	-	-3.66 (-10.74, 3.41)	-	**12.60 (0.38, 24.82)**
Isobutyric acid	-	-0.92 (-2.29, 0.44)	-	-0.08 (-1.60, 1.45)
Valeric acid	-	**-1.09 (-2.10, -0.08)**	-	-0.05 (-1.66, 1.57)
Isovaleric acid	-	-0.65 (-1.98, 0.68)	-	-0.35 (-2.07, 1.38)
Hexanoic acid	-	-0.35 (-0.83, 0.12)	-	-0.19 (-0.40, 0.03)

Table S13 [Footnote]: *All models adjusted for birth weight (kg), gestational age (weeks), maternal age (years), household income (<$20 000 per year vs. ≥ $20 000 per year), and duration of exclusive breastfeeding (months).*
Abbreviations: CI = confidence interval, SCFA = short chain fatty acid
